# Self–reported specific learning disorders and risk factors among Hungarian adolescents with functional abdominal pain disorders: a cross sectional study

**DOI:** 10.1186/s12887-020-02167-w

**Published:** 2020-06-06

**Authors:** János Major, Szilvia Ádám

**Affiliations:** 1 Károly Rácz School of PhD Studies, Semmelweis University, Üllői str. 26., Budapest, H-1085 Hungary; 2 HRC Bethesda Children’s Hospital, Bethesda str. 3., Budapest, H-1146 Hungary; 3 Faculty of Health and Public Services, Health Services Management Training Centre, Semmelweis University, Kútvölgyi str. 2., Budapest, H–1125 Hungary

**Keywords:** Abdominal pain, Epidemiology, Adolescents, Irritable bowel syndrome, Chronic pain

## Abstract

**Background:**

Despite its increasing clinical significance and diagnostic challenges, little is known about functional abdominal pain disorders (FAPDs) in Central-Eastern Europe. In this paper, the prevalence and potential sociodemographic correlates of FAPDs among Hungarian adolescents are explored.

**Methods:**

A cross-sectional, nationwide, questionnaire study in a representative sample of 657 adolescents has been conducted. With a response rate of 80.2%, 522/527 (99.1%) questionnaires were eligible for data analysis (*N* = 267, 51.1% girls, mean age 14.8, SD 2.4 years). The questionnaire included sociodemographic variables (age, sex, place of residence, marital status of the parents, family income, religion, educational level of parents), questions regarding self–reported specific learning disorders and the Questionnaire for Paediatric Gastrointestinal Symptoms Rome ΙΙΙ Edition.

**Results:**

The prevalence of FAPDs was 11.9% (*N* = 62). FAPDs were significantly associated with female sex. Living in a county town showed a negative correlation with FAPD. Adolescents with self–reported arithmetic learning disorders had an 8.7-fold likelihood of FAPD (OR, 8.7; 95% CI (3.5–21.9). Adolescent girls reported pain in all subtypes of FAPDs more frequently than adolescent boys except functional abdominal pain syndrome. The most prevalent FAPD was abdominal migraine (*N* = 32, 6.1%), followed by irritable bowel syndrome (*N* = 24, 4.6%).

**Conclusions:**

The prevalence of FAPDs in Hungary is similar to that reported worldwide, however, contrary to international data, abdominal migraine is the most frequently encountered FAPD in Hungary. In addition to well-known correlates of FAPDs, such as female sex and place of residence, arithmetic learning disorders have also been identified as correlating with the prevalence of FAPDs. Our results suggest culture-specific differences in the distribution of FAPDs, and confirm the significance of school performance indicators such as specific learning disorders as a correlate of FAPDs.

## Background

The prevalence of functional abdominal pain disorders (FAPDs) ranges between 6.3–27.3% among adolescents as defined by the Rome III criteria, which classify the disorders into 5 subtypes [1, 2]. FAPDs among adolescents are a global health challenge as they may adversely impact their quality of life and daily functioning; may lead to psychiatric co-morbidities and an increase in associated health-care costs; and may result in significant morbidity later in adulthood [1–3]. Abdominal pain in adolescents with FAPDs should be differentiated from conditions that require medical or surgical treatment. The most common organic conditions that may overlap with similar symptomatology are acute gastritis, cholecystitis, appendicitis, ovarian or testicular torsion, ovarian cysts etc. [4–6]. However, a recent study with strong statistical power pointed out that only a small proportion (5.8%) of those with non-specific abdominal pain are diagnosed with underlying bowel pathology in long-term [7]. Therefore, early diagnosis and thorough understanding of the potential risk factors of FAPDs are paramount in order to provide effective treatment, improve outcomes, and reduce ensuing healthcare costs.

The pathogenesis of the disorder is multifactorial. The biopsychosocial model describes several risk factors (genetic, physiological, psychological and social) leading to visceral hypersensitivity, altered motility and FAPD [8, 9]. Studies on sociodemographic risk factors such as age, place of residence, marital status, educational level of the parents and socioeconomic status of the family have yielded controversial results [1, 10–26]. Wider social and educational factors, such as school performance and relations with peers and teachers play a unique role in developing chronic abdominal pain in children [13]. Specific learning disorders (SLD) such as writing, arithmetic, and reading impairment, are frequent in the general population (2–10%) and they markedly influence school performance, leading to emotional burden and stress in school. However, the relationship between FAPDs and SLDs has not been studied [27]. Similarly, associations between religiosity and FAPDs were not studied in the adolescent cohort; however, it may have a positive impact on pain coping strategies, as demonstrated in adult patients [28].

Despite the clinical significance of FAPDs and emerging associations with various socio-demographic variables, no information is available about the prevalence and potential risk factors of FAPDs among adolescents in Hungary. The country is undergoing radical societal changes and transformation of the educational system characterised by high psychosocial stress, which has been shown to influence family life as well as the health of adolescents [29]. Therefore, the objective of this research was to explore the prevalence and potential socio-demographic correlates of FAPDs among adolescents in Hungary, with the ultimate aim at supporting clinical decision-making and effective management of patients.

## Methods

A national cross-sectional study was conducted in April 2016. Adolescent school children in grades 5–12 (every 5th pupil from each class) and their parents from 22 schools in all of the 8 regions nationwide were randomly selected, to obtain a representative cohort for all school grades, sex, and regions. The most recent Hungarian Statistical Yearbook of Education 2013/2014 was used, to ensure that the sample has matching socio-demographic characteristics to those of the normative population [30]. The minimal sample size (*N* = 602) was determined based on previous epidemiological studies on the topic, which took into consideration a total prevalence of 10%, 95% confidence interval, 5% degree of absolute precision, and 20% attrition [13–16].

All adolecents who were selected by the randomization process (incl. missing students) were included in the study. The lack of data on sex and answers necessary to diagnose a specific FAPD was excluded from the analysis.

### Setting

Printed questionnaires were mailed to each school. Teachers asked the subjects to complete questionnaires during school-time and at home, within 2 weeks. The questionnaires were anonymous. Consent forms were sent to the parents and only children who obtained informed consent from their parents were allowed to participate in the study.

### Main outcome measures

#### Primary outcomes measures

The validated Questionnaire for Paediatric Gastrointestinal Symptoms Rome ΙΙΙ Edition (QPGS-III) was used to assess abdominal pain [2]. The questionnaire categorizes abdominal pain into five subtypes: functional dyspepsia (FD), irritable bowel syndrome (IBS), abdominal migraine (AM), functional abdominal pain (FAP) and functional abdominal pain syndrome (FAPS). The QPGS-III was translated into Hungarian by 3 researchers and then back-translated into English by an independent bilingual professional translator. Additionally, a native English–speaking professional reviewed the back-translated version and compared it with the original. Discrepancies were resolved by team discussion and adjustment to the translation. This translation was allowed but not authorized by the Rome Foundation.

#### Secondary outcome measures

Subjects were asked about their age, sex, place of residence and region (geographical area), as well as the marital status of their parents. If the parents were divorced, participants were asked about which parent they lived with. A question relating to participants’ religion was also included: whether they belonged to a church that they regularly visited. In a separate question, the prevalence of previously diagnosed SLDs was asked. If participants answered positively, further questions explored the type of learning disorder, such as writing, arithmetic, reading, or other learning disorders.

Additionally, parents were asked about the estimated total monthly income of the family per capita (1: under 100 €; 2: 100–200 €; 3: 200–300 €; 4: 300–600 €; 5: above 600 €). Parents’ highest attained educational level was also explored on a 5-point scale (1: less than 8 years of primary education; 2: primary school completion (8 years of education); 3: secondary school completion (12 years of education); 4: advanced level grade (13 years of education) 5: higher education degree (14 years or more of education).

### Data analysis

Descriptive statistics for frequency counts were deployed to explore the prevalence of FAPDs and their types; Mann-Whitney U test, χ2 test, and Fisher test were used to evaluate differences in the prevalence of FAPDs between various study groups, and categorical variables. Odds ratios were calculated to assess the strength of the relationships between the variables. Missing data was excluded from the further analyses. Data was analysed by SPSS software version 24.0 (IBM SPSS Statistics, IBM Corporation, Chicago, IL) and *p* < 0.05 was considered as statistically significant.

## Results

Of the 657 questionnaires distributed to the schools, 527 were returned (response rate of 80.2%). We excluded 5 questionnaires due to missing values, which left 522 questionnaires (79.5%) for data analysis (*N* = 267, 51.1% girls, mean age 14.8, SD 2.4 years).

The prevalence of FAPDs was high among Hungarian adolescents (*N* = 62, 11.9%). In our sample, the most prevalent FAPD was abdominal migraine (*N* = 32, 6.1%) followed by irritable bowel syndrome (IBS) (*N* = 24, 4.6%). Functional abdominal pain syndrome (FAPS) was more prevalent among male adolescents, all other FAPDs were more frequent among females (Table 1). We identified a significantly higher prevalence of AM among female adolescents.

**Table 1:** The prevalence of FAPDs among female and male adolescents (*N* = 522)

Type of FAPD	Male N (% of the total male)	Female N (% of the total female)	*p*
Functional dyspepsia (FD)	0 (0.00)	4 (1.5)	NS✺
Irritable bowel syndrome (IBS)	10^ρ^ (3.9)	14^a^ (5.2)	NS ✣
Abdominal migraine (AM)	8 (3.1)	24 (9.0)	*p* = 0.002 ✣
Functional abdominal pain (FAP)	2 (0.8)	7 (2.6)	NS ✺
Functional abdominal pain syndrome (FAPS)	2 (0.8)	1 (0.4)	NS ✺
Total (% of the total sex)	18 (7.1)	44 (16.5)	*p* = 0.001✣

*NS* nonsignificant

ρ 4 adolescents also reported AM

^a^6 adolescents also reported AM

✣ significance of chi-square test

✺ significance of the 2–sided fisher’s exact test

**Fig. 1:**
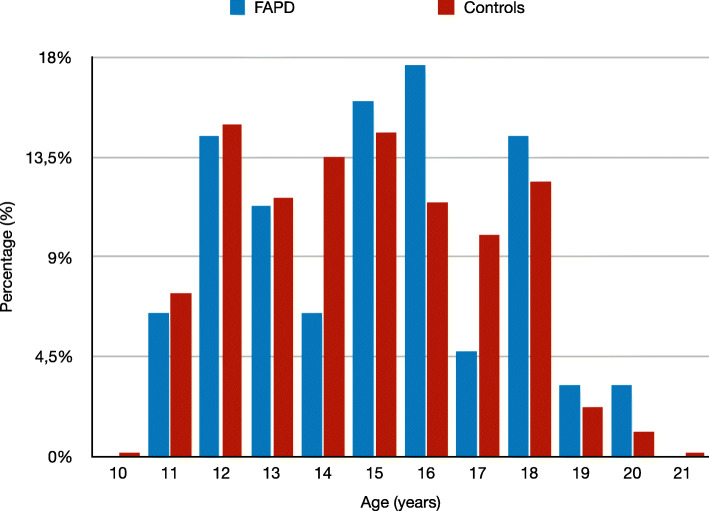
Age distribution of FAPDs and controls

The prevalence of FAPDs was the highest in the 16-year-old cohort (Fig. 1), however, when age was compared to the controls, no statistical difference in the age distribution was found.

Of all other socio-demographic variables, place of residence emerged as a significant correlate of FAPDs. Adolescents who lived in county towns reported significantly lower prevalence of FAPDs versus those who lived elsewhere. We identified no significant associations between FAPDs and the regions (geographical areas) (Table 2). We found no further socio-demographic correlates of FAPDs (Table 3).

**Table 2:** Place of residence in adolescents with functional gastrointestinal disorders and controls (N = 522)

	Adolescents with FAPDs N (%)	Controls N (%)	***p***
**Place of residence**			
Village	17 (27.4)	105 (22.8)	NS✣
Small town	25 (40.3)	192 (41.7)	NS✣
County town	5 (8.0)	91 (19.8)	*p* = 0.03✺
Capital	14 (22,6)	69 (15)	NS✣
Missing	1 (1.6)	3 (0.7)	
**Region of residence**			
Budapest	14 (22.6)	68 (14.8)	NS✣
Pest county	8 (12.9)	77 (16.7)	NS✣
Northern-Hungary	5 (8.1)	62 (13.5)	NS✺
Northern-Great Plain	8 (12.9)	62 (13.5)	NS✣
Southern-Great Plain	7 (11.3)	59 (12.8)	NS✣
Southern-Transdanubia	4 (6.5)	43 (9.3)	NS✺
Central-Transdanubia	10 (16.1)	53 (11.5)	NS✣
Western-Transdanubia	6 (9.7)	36 (7.8)	NS✣
Missing	0 (0.0)	0 (0.0)	

*NS* Nonsignificant

✣ significance of chi-square test

✺ significance of the 2–sided fisher’s exact test

**Table 3:** Socioeconomic and family characteristics in adolescents with functional gastrointestinal disorders and controls (*N* = 522)

	Adolescents with FAPDs N (%)	Controls N (%)	***p***
**Mother’s educational level (SD)**			
Less than year 8	1 (1.6)	5 (1.1)	NS✺
Primary school	3 (4.8)	24 (5.2)	NS✺
Secondary school	14 (22.6)	103 (22.4)	NS✣
Advanced level	25 (40.3)	140 (30.4)	NS✣
Higher education	15 (24.2)	151 (32.8)	NS✣
Missing	4 (6.5)	37 (8.0)	
**Father’s educational level (SD)**			
Less than year 8	1 (1.6)	3 (0.7)	NS✺
Primary school	7 (11.3)	29 (6.3)	NS✣
Secondary school	17 (27.4)	122 (26.5)	NS✣
Advanced level	13 (21.0)	124 (27.0)	NS✣
Higher education	12 (19.4)	103 (22.4)	NS✣
Missing	12 (19.4)	79 (17.2)	
**Mean income of the family (SD)**			
Under 100 €	4 (6.5)	29 (6.3)	NS✺
100–200 €	14 (22.6)	113 (24.6)	NS✣
300–400 €	20 (32.3)	127 (27.6)	NS✣
500–600 €	10 (16.1)	86 (18.7)	NS✣
Above 600 €	6 (9.7)	30 (6.5)	NS✣
Missing	8 (12.9)	75 (16.3)	
**Parents’ marital status**			
Married	39 (62.9)	308 (67.0)	NS✣
Divorced	22 (35.5)	146 (31.7)	NS✣
Missing	1 (1.6)	6 (1.3)	
**Placed with/living with**			
Father	1 (4.5)	19 (13.0)	NS✺
Mother	19 (86.4)	122 (83.6)	NS✣
other relatives	2 (9.1)	4 (2.7)	NS✺
Missing	0 (0.0)	1 (0.7)	
**Member of a religious community**	14 (22.6)	79 (17.4)	NS✣
Missing	0	7 (1.5)	

*NS* nonsignificant

✣ significance of chi-square test

✺ significance of the 2–sided fisher’s exact test

The relationship between self–reported SLDs, a proxy of performance at school, and FAPDs was also explored. Our results showed that adolescents with previously diagnosed SLDs have a 2.2-fold likelihood of having FAPD, which is significantly higher than subjects without FAPD. In our study, SLD with impaired arithmetic skills was the most prevalent learning disorder, and it was associated with an 8.7-fold likelihood of FAPDs (Table 4).

**Table 4:** Prevalence of SLDs and their relationship with FAPDs among adolescents with FAPDs and controls (*N* = 522)

	Adolescents with FAPDs N (% of adolescents with FAPDs)	Controls N (% of controls)	***p***	Odds ratio (95% CI)
**SLD**	12 (19.4)	46 (10.0)	*p* = 0.03✣	2.2 (1.1–4.4)
No SLD	49 (79.0)	406 (88.3)		
Missing	1 (1.6)	8 (1.7)		
**Distribution of SLD**				
Writing	0 (0.0)	9 (2.0)		
Reading	0 (0.0)	7 (1.5)		
Arithmetic	10 (16.1)	10 (2.2)	*p* = 0.00✣	8.7 (3.5–21.9)
Other	1 (1.6)	9 (2.0)	NS✺	0.8 (0.1–6.6)
Complex	1 (1.6)	14 (3.0)	NS✺	0.5 (0.1–4.0)
Missing	1 (1.6)	7 (1.5)		

*NS* nonsignificant

✣ significance of chi-square test

✺ significance of the 2–sided fisher’s exact test

## Discussion

In this community based representative study among Hungarian adolescents, we identified a prevalence of 11.9% for FAPDs. Our results are in line with those reported by a number of epidemiological studies, which demonstrated a mean total prevalence of FAPDs of 12.48% (range of 6.3–27.3%) [1, 11–23]. Two studies from Europe with robust methodologies also reported a prevalence of FAPDs of around 10% (9.2 and 14.9%) [11, 12]. We hypothesize that the reasons for the wide difference in prevalence rates reported by these studies might be due to different cultural, economic and social factors (e.g. school system), as postulated by other researchers [31]. Furthermore, we expect that differences in methodologies can also contribute to this wide range of results. Namely, most of the surveys were conducted in a smaller area, or even in one city of the given country, in a limited number of schools, which could further bias the results.

In almost all studies, IBS has been shown to be the most prevalent FAPD in the adolescent age group followed by FAP [1, 11–23]. In our sample, AM was more frequent than IBS. Our findings appear to confirm those of Bouzios et al., the most recent study of MEAP working group, and Nelissen et al. [11, 12, 23]. We postulate that the different cultural milieux could influence the distribution of the different subtypes of FAPDs in a given population, however, further cross-cultural studies are needed to investigate the causes of this phenomenon.

On the other hand, it is well-accepted that the prevalence of AM is overestimated using the Rome III criteria vs the Rome IV or Rome II criteria. This might have led to the overestimation of the prevalence of AM in our sample [32].

We found a higher prevalence of FAPDs among female vs. male adolescents. Our findings confirm results from previous research on the relationship between FAPDs and sex. Our finding of sex differences in the prevalence of FAPDs could be explained by sex differences in pain perception and processing. Evidence suggests that female adolescents exhibit enhanced central pain sensitivity possibly also due to sex hormonal effects [33].

FAPD was more prevalent among female adolescents in all subtypes except FAPS. This supports our clinical impression that the most disabling cases of FAP are among adolescent boys. In a study from Sri Lanka, FAP was also found to be more prevalent in boys, but the authors did not publish any data about the prevalence of FAPS [31]. Studies in Sri Lanka, Japan, Greece and in other Mediterranean countries, however, demonstrated female predominance in all subtypes of FAPDs [11–13, 15].

We found no differences in the prevalence of FAPDs among various age cohorts in this study. These results are in line with those of the Mediterranean–European Area project on functional gastrointestinal disorders (MEAP) [12]. Similarly, the meta–analysis of Korterink et al. compared pooled data from children below and above the age of 12 and confirmed no difference in the two age cohorts [1]. Furthermore, Sri Lankan researchers described a peak in the prevalence of FAPDs at ages 14–16, which is consistent with our data [14].

Our findings demonstrated that living in towns (defined as a population of 50–200,000 inhabitants) had an inverse relationship with FAPDs. Our findings add further data to a very small body of evidence presented only in three studies on non-representative samples, about the associations between the type of the settlements and FAPDs [11, 13, 19].

We found no difference in the prevalence of FAPDs among geographical areas with low and high economic status, despite the fact that there is more than a two–fold difference between the poorest and the richest part of the country according to data on gross domestic product per capita, as reported by the National Statistical Agency on 2015 [34].

We expected a lower prevalence of FAPDs in higher income families and in families where parents had higher educational levels, however, we could not confirm our hypothesis. To our knowledge, our study is one of the first to explore the relationship between different geographical areas, family income, educational level of the parents and FAPD, according to Rome III. The studies assessing socio–economic status by other methodologies demonstrated either no difference, or a higher probability of any FGID (but not with FAPDs, per se) with lower socioeconomic status [11, 13, 19]. Family stress has been shown to impact the development of abdominal pain as demonstrated by a recent meta-analysis [1]. In our study, we did not find an association between divorce or marital status and the prevalence of FAPDs. Our results confirm those reported in studies from Sri Lanka, Colombia, El Salvador and Argentina [13, 16, 18, 23]. On the other hand, Bouzios et al. found that single parent households were associated with a higher probability of any FGID (but not specifically FAPDs) [11]. We hypothesise that the pathogenetic factor in the development of FAPDs could be family stress and that divorce or single parent household alone may not be a risk factor [1].

Membership of a religious community did not significantly influence the prevalence of FAPDs in our sample. To our knowledge, no other studies have examined religiosity in connection with FAPDs. Research by German scientists about chronic pain among patients in adult out–patient setting identified associations between spirituality/religiosity, positive appraisals and internal adaptive coping strategies with pain [28]. Further research is needed to understand the role of spirituality in a population of children and adolescents.

SLDs were identified as a new possible correlate of FAPDs. The role of learning disorders in the pathogenesis of FAPDs has not been studied before. It may be suggested that this relationship can be explained by higher school stress and emotional burden due to SLDs, which in turn may support the development of FAPDs. Indeed, school stress and emotional burden have been implicated in the maintenance of FAPDs [13].

In our evaluation, the present study has several strengths. This is the first survey that reports epidemiological data about FAPDs from Central and Eastern Europe using the Hungarian version of Rome III questionnaires, which allows for more appropriate cross–cultural comparison. In addition, our study used a representative sample for various socio-economic variables to explore probable correlates of FPADs. Finally, this is the first study that explored the possible association between SLDs and FAPDs. Another of our study is the use of the child–report methodology, as parental reports often underestimate the prevalence of these disorders. Our methodology allows for a more accurate assessment of the prevalence of FAPDs [1].

Our study also has some limitations. The cross-sectional setting does not allow for cause–and–effect conclusions. We did not include all school-types from all regions and did not perform a detailed work–up regarding specific learning disorders, which further limits interpretation of our results. Participants did not receive physical examinations, which may distort some of our findings.

## Conclusions

Our results provide important insights into potential risk factors of FAPDs and uncover likely associations between FAPDs and SLDs, an indicator of school performance. These results may add further impetus to cross-cultural clinical research in the field, and aid clinical decision-making and diagnosis. Further research is needed to explore the role of sex, place of residence and SLDs in the pathogenesis and evolution of abdominal pain among adolescents.

## Data Availability

The datasets supporting the conclusions of this article are included within the article.

## Abbreviations

AM: Abdominal migraine
FAPD: Functional abdominal pain disorder
FAP: Functional abdominal pain
FAPS: Functional abdominal pain syndrome
FD: Functional dyspepsia
FGID: Functional gastrointestinal disorder
IBS: Irritable bowel syndrome
MEAP: Mediterranean–European Area Project
SLD: Specific learning disorder
QPGS-III: Questionnaire for Paediatric Gastrointestinal Symptoms Rome ΙΙΙ Edition
